# COVID-19-Associated Pneumomediastinum and Pneumothorax: A Case Series

**DOI:** 10.7759/cureus.17715

**Published:** 2021-09-04

**Authors:** Ankita Kabi, Nidhi Kaeley, Takshak Shankar, Shrirang Joshi, Pradeep Kumar Roul

**Affiliations:** 1 Emergency Medicine, All India Institute of Medical Sciences, Rishikesh, Rishikesh, IND; 2 Radiology, All India Institute of Medical Sciences, Rishikesh, Rishikesh, IND

**Keywords:** patient self-induced lung injury, pneumomediastinum, pneumothorax, covid-19, macklin phenomenon

## Abstract

Coronavirus disease-19 (COVID-19) causes mild to moderate illness in most patients but in some cases a severe illness may manifest. Such patients usually present with hypoxaemic respiratory failure due to acute lung injury caused by a viral infection and host-mediated cytokine storm. The characteristic radiographic findings are ground-glass opacities with consolidation in posterior basal areas of bilateral lungs and rarely pneumothorax (PTX) and pneumomediastinum (PM). The incidence of these findings was notably higher in the second wave of the pandemic in India in 2021 as compared to the first wave in 2020. The etiopathogenesis of this life-threatening condition can be due to Macklin phenomenon post-cytokine-mediated diffuse alveolar injury, patient self-inflicted lung injury (P-SILI), and barotrauma in mechanically ventilated patients. The presence of pneumomediastinum is associated with higher mortality rates, prolonged intensive care unit (ICU) stays making it a poor prognostic marker. There is no consensus regarding its management in COVID-19 patients although both aggressive and conservative strategies have been tried.

## Introduction

Pneumomediastinum (PM) and pneumothorax (PTX) is a rare complication seen in patients afflicted with a severe form of coronavirus disease-19 (COVID-19). It can be spontaneous, attributed to cytokine-mediated diffuse alveolar injury with the well-known Macklin phenomenon, or secondary to barotrauma and volutrauma inpatient self-inflicted lung injury (P-SILI) or mechanical ventilation. These changes appear to be specific to COVID-19 which may help to explain the higher incidence of pneumomediastinum and pneumothorax in COVID-19 compared to other types of pneumonia at equal severity [1]. This trend of the increased incidence in the second epidemic wave signifies the pathomorphosis of the disease. The pointers of PM and PTX in such patients are rapid progression or acute presentation of dyspnea, chest pain, tachycardia, tachypnea, subcutaneous emphysema, and hypoxemia. These patients should be immediately assessed to rule out PM and PTX. We report four cases of PM with PTX who had acute respiratory distress syndrome (ARDS) due to COVID-19 out of which two were managed conservatively while pigtail decompression was done in the other two patients. The pneumomediastinum was successfully managed in all the patients but three patients succumbed to multiple organ dysfunction syndromes (MODS) while one patient is still recovering in the intensive care unit.

## Case presentation

Case 1

**Figure 1:**
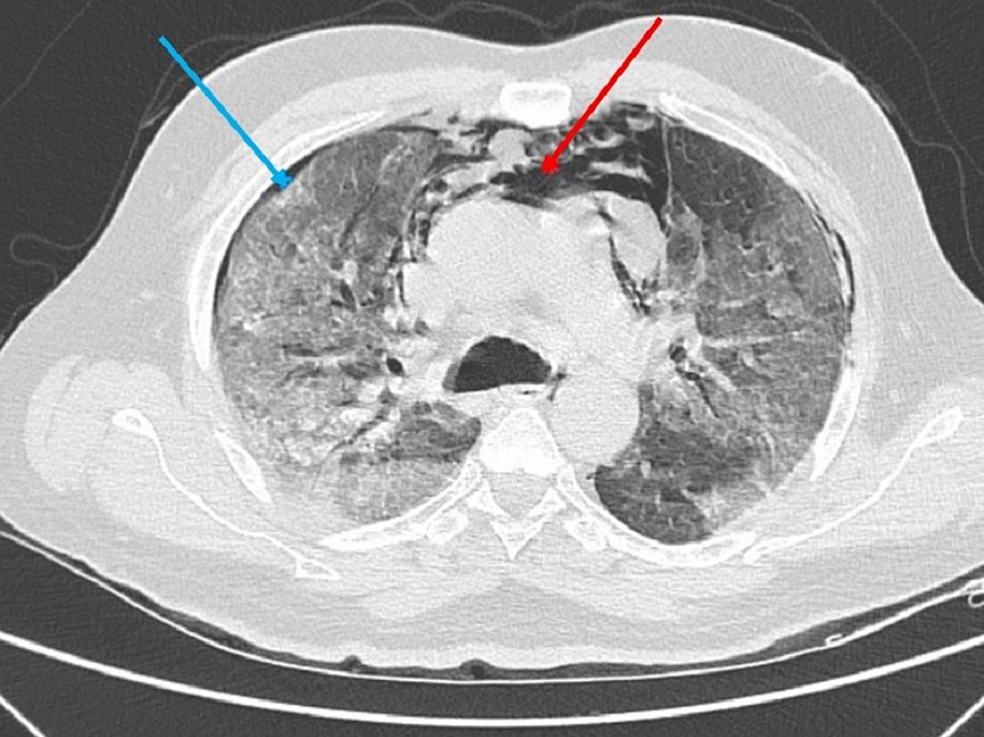
High-resolution computed tomography of the thorax-axial section showing pneumomediastinum (red arrow) and pneumothorax (blue arrow).

**Figure 2:**
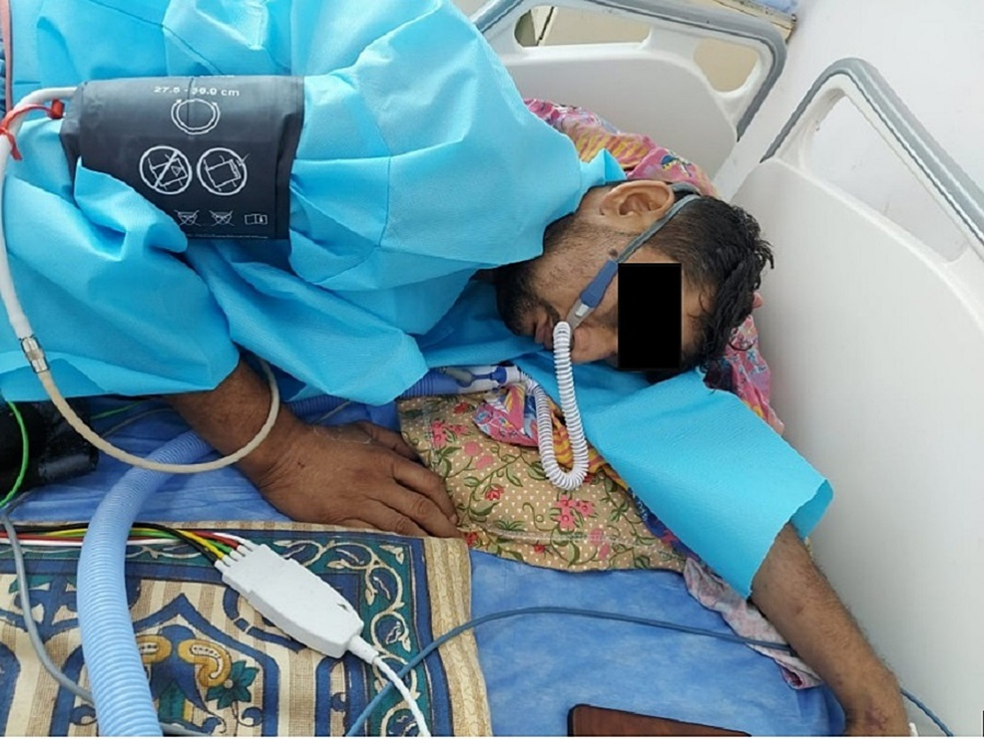
Patient put on high flow nasal cannula.

A 40-year-old male with no known comorbidities presented to the emergency department with fever and breathlessness of four days duration. On arrival, his oxygen saturation on room air (SpO2) was 40% with a respiratory rate of 40 per minute. He was administered oxygen through a non-rebreather mask (NRBM) at a flow of 15 liters per minute (L/min) and positioned prone following which his peripheral oxygen saturation (SpO2) improved to 94%. A high-resolution computed tomography scan (HRCT) of thorax revealed COVID-19 Reporting and Data System (CORADS) 5 with a computed tomography severity score (CTSS) of 15/25. His real-time reverse transcription-polymerase chain reaction (rRT-PCR) turned out to be positive for severe acute respiratory syndrome coronavirus 2 (SARS-Cov-2). On day 3 of admission in the intensive care unit (ICU), he was put on non-invasive ventilation (NIV) with fractional inspired oxygen concentration (FiO2) 70%, pressure support (PSV) of 5 cm H2O, and positive end-expiratory pressure (PEEP) of 6 cm H2O due to increased work of breathing. After few hours, he developed subcutaneous emphysema over his neck. Immediately a bedside lung ultrasonography was done which showed a bar code sign in the right upper zone of the lung signifying a pneumothorax. His repeat HRCT revealed a CTSS of 24/25 with gross pneumomediastinum and bilateral minimal pneumothorax (Figure 1). A pigtail catheter was inserted in the mediastinum. The patient’s respiratory rate decreased to 28 per minute and hence he was put on high-flow nasal cannula (HFNC) at FiO2 of 60% with a flow of 60 L/min (Figure 2). The pneumomediastinum resolved over 12 days during which his oxygen delivery device was weaned to a face mask. Despite initial improvement in his clinical condition, he later developed fungal pneumonia with multiple organ dysfunction syndrome (MODS) and expired on day 28.

Case 2

**Figure 3:**
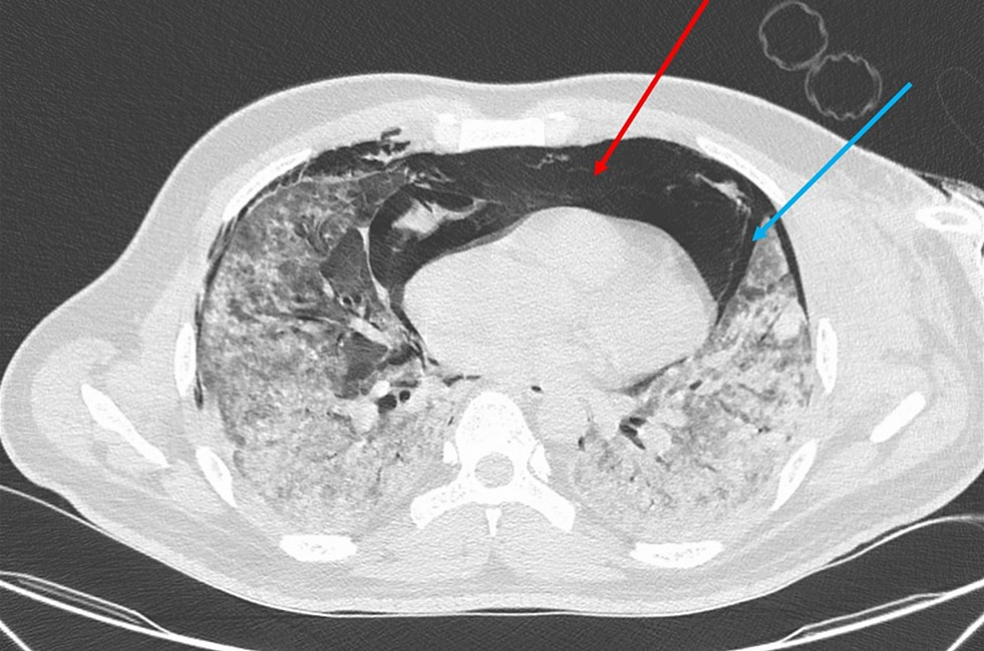
High-resolution computed tomography of the thorax-axial section showing pneumomediastinum (red arrow) and pneumothorax (blue arrow).

**Figure 4:**
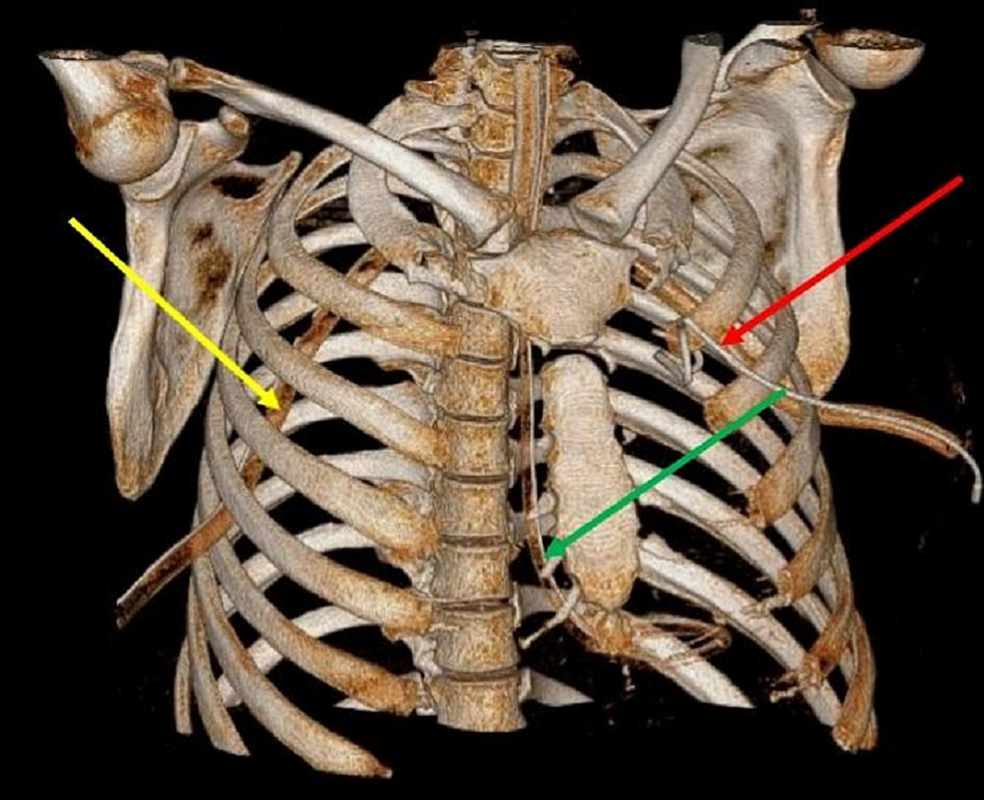
Volume rendered technique (VRT) image of the chest wall showing pigtail (red arrow), Ryle’s tube (green arrow) and intercostal drainage tube (yellow arrow).

**Figure 5:**
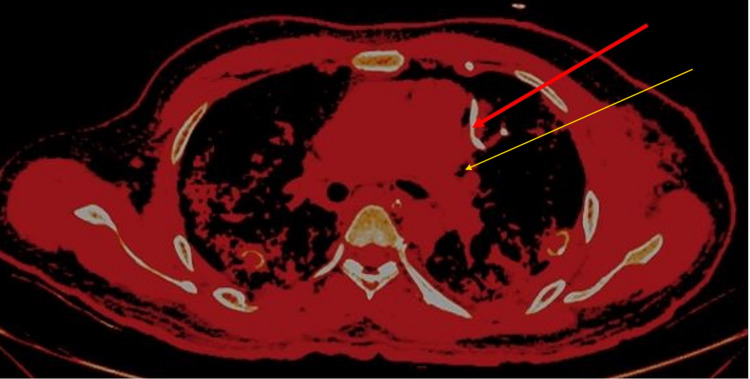
Volume rendered technique (VRT) image of the thorax-axial section showing tip of the pigtail catheter in the mediastinum (red arrow). Also note the residual pneumomediastinum (yellow arrow).

**Figure 6:**
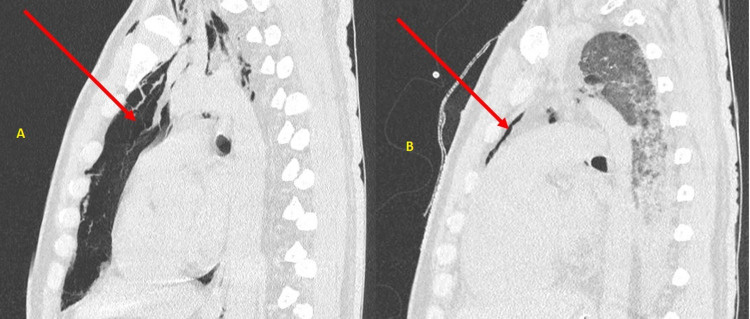
(A) High-resolution computed tomography of the thorax – sagittal section showing pneumomediastinum (red arrow). (B) High-resolution computed tomography of the thorax – sagittal section showing reduction in the size of the pneumomediastinum following pigtail insertion (red arrow).

A 34-year-old male came to the emergency department complaining of cough for 10 days, fever, and breathlessness for eight days. He had no known comorbidities. He smoked two cigarettes/day for the last five years. On arrival, he was tachypneic with a respiratory rate of 45/min and peripheral oxygen saturation (SpO2) of 70% on room air. rRT-PCR testing done for SARS-Cov-2 was positive. He was put on oxygen support at 15 L/min via a non-rebreather mask (NRBM) initially, following which he was maintaining a peripheral oxygen saturation (SpO2) of 93% and respiratory rate of 26/min. The initial high-resolution computed tomography scan (HRCT) of the thorax was suggestive of COVID-19 Reporting and Data System (CORADS) 6, with a computed tomography severity score (CTSS) of 38/40. He also had 3.6 mm left-sided pneumothorax. On day 15, his respiratory distress worsened for which non-invasive ventilation (NIV) with FiO2 of 50%, PEEP of 8 cm H2O, and pressure support (PSV) of 6 cm H2O was started. Remdesivir-200 mg IV OD for the first day and 100 mg IV OD for four days and steroids-dexamethasone 6 mg IV OD for 14 days were given to the patient as per the institutional protocol. On day 18 of admission, he went into cardiac arrest. Return of spontaneous circulation (ROSC) was achieved after three cycles of cardiopulmonary resuscitation (CPR). He was intubated and put on lung-protective ventilation as per the ARDS net protocol [2]. An HRCT thorax done the same day was suggestive of a massive pneumomediastinum of 5 cm thickness and minimal bilateral pneumothorax (Figure 3). Intercostal drainage (ICD) tube in the bilateral chest was inserted and a pigtail was inserted to decompress the pneumomediastinum (Figure 4). Follow-up HRCT thorax on the second day was suggestive of a significant reduction in pneumomediastinum (Figure 5) and the pigtail was removed on day 20. Figure 6 compares the size of the pneumomediastinum before and after pigtail insertion. His ICD tubes were removed subsequently. The patient has been decannulated and is currently recovering on oxygen support.

Case 3

**Figure 7:**
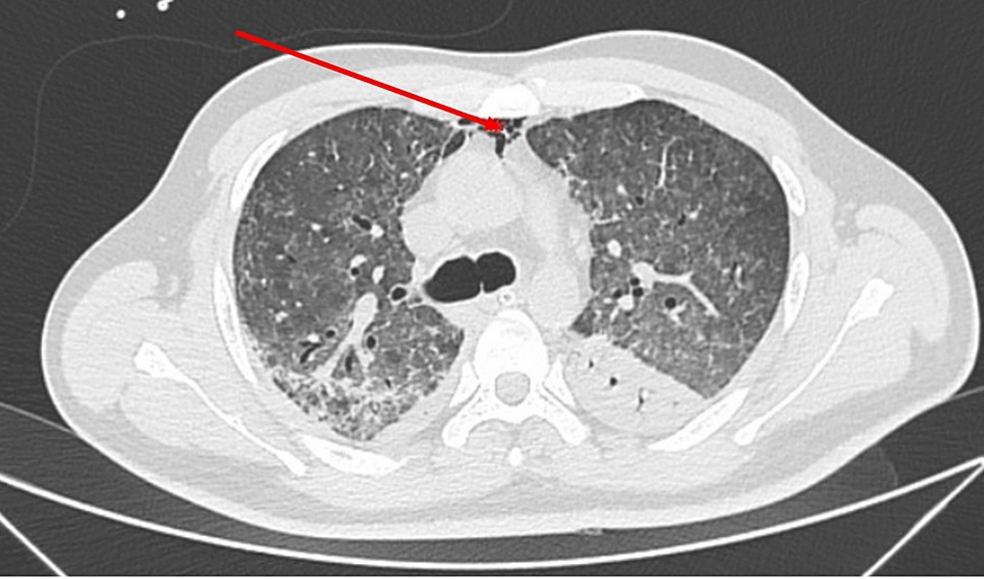
High-resolution computed tomography of the thorax-axial section showing pneumomediastinum (red arrow).

A 29-year-old male came to the emergency department complaining of high-grade fever for seven days, dry cough, and breathlessness for five days. His rapid antigen test (RAT) was positive for SARS-Cov-2. He had tachycardia, a SpO2 of 70% on room air, and a respiratory rate of 50/min. Due to his increased work of breathing, NIV was initiated with FiO2 of 80%, PSV of 6 cm H2O, and PEEP of 12 cm H2O. The pharmacological treatment was started as per the institutional protocol. On day 13 of admission, he had an episode of chest pain following which he became hemodynamically unstable with rapid desaturation. He was immediately intubated and put on pressure control mode ventilation following the ARDS net protocol [2]. An HRCT thorax with pulmonary angiography done the same day suggested a pneumomediastinum of 23 mm thickness (Figure 7). His CTSS was 34/40. Follow-up CT scans showed mild reduction in the same and the pneumomediastinum resolved by the third day. However, the patient’s clinical condition continued to deteriorate and he expired on day 23.

Case 4

**Figure 8:**
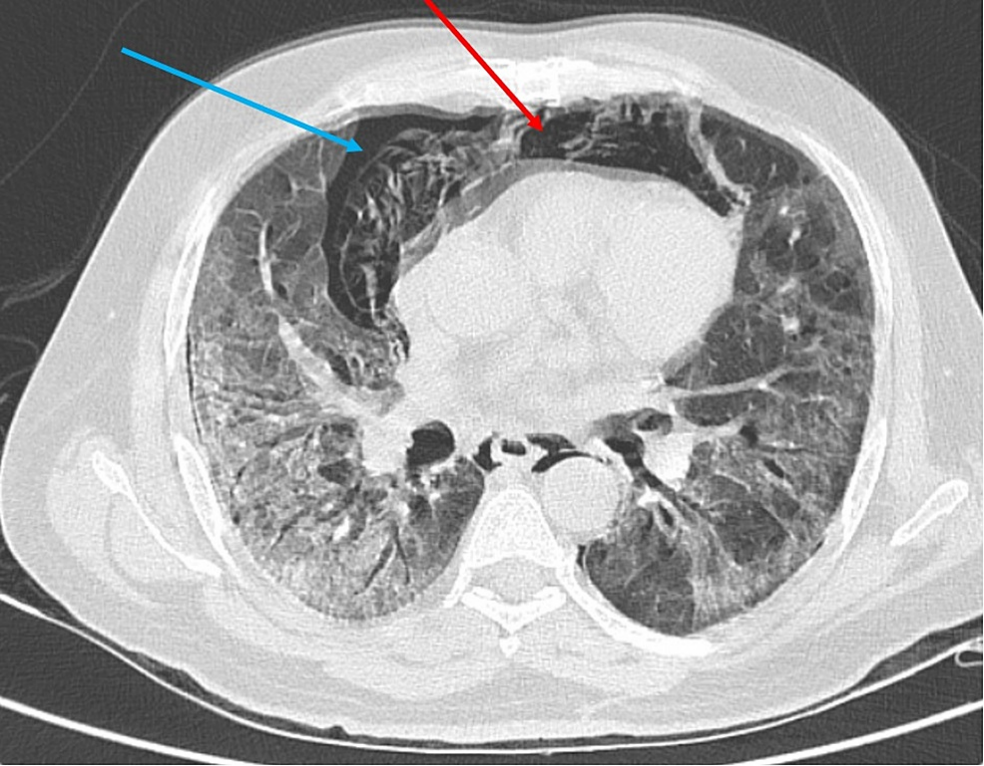
High-resolution computed tomography of the thorax-axial section showing pneumomediastinum (red arrow) and pneumothorax (blue arrow).

A 46-year-old male came to the emergency department complaining of high-grade fever for eight days, breathlessness for one day. He denied any addictions. His past medical history was significant for pulmonary tuberculosis 10 years back, for which he had taken complete treatment. He was also partially immunized against COVID-19 having received the first shot of the COVISHIELDTM vaccine around 20 days before. On arrival, he was tachycardic and hypoxic with a pulse rate of 100/min, blood pressure of 130/90 mm Hg, a Spo2 of 88% on room air and respiratory rate of 35/min. An rRTPCR test done for SARS-Cov-2 was positive. He was put on oxygen therapy at 10 L/min through NRBM following which his saturation improved to 92%. Steroid therapy was started for the patient as per the institutional protocol. On day 9 of admission, the patient desaturated. NIV trial was given to the patient but it failed. The patient was intubated and put on lung-protective ventilation as per the ARDS net protocol [2]. An HRCT of the thorax with pulmonary angiography was suggestive of gross pneumomediastinum of 5 cm thickness, bilateral minimal pneumothorax, and pulmonary thromboembolism involving a segmental and subsegmental branch of the right posterior basal segment (Figure 8). Therapeutic dose anticoagulation was started for the pulmonary embolism. ICD tubes were inserted for the pneumothoraces. A follow-up HRCT of the thorax showed a reduction in the size of the pneumomediastinum and hence the pigtail insertion was deferred. The patient’s clinical condition continued to worsen and he expired on day 13.

All the four cases have been summarized in Table 1.

**Table 1:** Patient characteristics.

	Patient 1	Patient 2	Patient 3	Patient 4
Clinical Profile				
1. Age	40	34	29	46
2. Gender	Male	Male	Male	Male
3. Symptoms				
a. Fever	Yes	Yes	Yes	Yes
b. Cough	No	Yes	Yes	No
c. Chest Pain	No	No	No	No
d. SOB	Yes	Yes	Yes	Yes
4. Previous lung disease	No	No	No	Yes
5. Hypoxemia	Yes	Yes	Yes	Yes
6. Subcutaneous Emphysema	Yes	Yes	No	No
Investigations				
1. Neutrophil-Lymphocyte Ratio	45	44	26	29
2. D-Dimer (mcg/ml)	5	8.8	2.7	>20
3. C-Reactive Protein (mg/L)	148	59.56	Not Ordered	59.58
4. Procalcitonin (ng/ml)	0.27	2.4	3.47	0.14
5. Chest X-ray	Yes	Yes	Yes	Yes
6. High-Resolution Computed Tomography (HRCT) thorax				
a. Computed tomography severity score (CTSS)	37/40	40/40	34/40	37/40
b. Pneumothorax	Yes	Yes	No	Yes
c. Pneumomediastinum	Yes	Yes	Yes	Yes
Initial Ventilator Mode	BiPAP	BiPAP	BiPAP	BiPAP
1. Positive End-Expiratory Pressure (PEEP)	6	8	12	10
2. Pressure Support (PSV)	5	6	6	6
3. PaO2/FiO2 Ratio	77	184	100	67
Ventilator Mode after Pneumomediastinum	BiPAP	AC-VC	AC-VC	BiPAP
1. Tidal Volume		350	360	
2. Positive End-Expiratory Pressure (PEEP)	5	5	6	6
3. Pressure Support (PSV)	0	5	6	6
4. PaO2/FiO2 Ratio	60	105	80	49
Treatment				
1. Remdesivir	Yes	Yes	Yes	No
2. Antibiotics	Yes	Yes	Yes	Yes
3. Azithromycin	Yes	Yes	Yes	Yes
4. Steroids	Yes	Yes	Yes	Yes
5. Therapeutic anticoagulation	No	Yes	Yes	Yes
6. Tocilizumab	No	No	No	No
7. Convalescent Plasma	No	No	No	No
8. Proning	Yes	Yes	Yes	Yes
9. Drainage	Yes	Yes	No	No
Outcome				
1. Ventilator days	20	30	24	8
2. Ventilatory day of Pneumomediastinum	3	3	12	4
3. Tracheostomy	Yes	Yes	No	No

## Discussion

COVID-19 is a highly contagious infectious disease that has resulted in over 300,000 deaths in India alone as of June 2021 [3]. The pulmonary system is commonly affected with the most frequent clinical manifestations of fever, cough, and dyspnea. The pulmonary complications range from acute respiratory distress syndrome to pneumothorax. The typical radiological findings in patients with COVID-19 are ground-glass opacities and/or consolidations with subpleural distribution, mostly affecting bilateral lower lobes and posterior segments of lungs. Other rare findings include pneumothorax, pneumomediastinum, and pneumopericardium. The reported incidence of PM is 4% among patients with ARDS. PM also serves as a predictive factor for PTX in these patients [4].

PM can be spontaneous or secondary to mechanical ventilation (MV) in COVID-19 patients. The pathophysiology of spontaneous PM is attributed to the Macklin phenomenon where a large pressure gradient between the marginal alveoli and the lung interstitium leads to alveolar rupture causing an air leak into the surrounding bronchovascular sheath [5]. The released air can track to the subcutaneous tissue causing subcutaneous emphysema, to the pericardial tissue causing pneumopericardium, to the pleural cavity causing pneumothorax or the spinal cord causing pneumorrhachis [6]. Non-intubated spontaneously breathing patients have a predilection for P-SILI owing to various factors - increased stress and strain of the lung, inhomogeneous distribution of ventilation leading to pendelluft creation, changes in lung perfusion leading to ventilation-perfusion mismatch, and patient-ventilator asynchronies during NIV. The P-SILI together with the COVID-19-induced cytokine storm results in a diffuse alveolar injury making the alveoli more prone to rupture [4,7]. Our three patients who were initially managed on oxygen therapy using NRBM and proning, developed acute onset of breathlessness for which NIV was initiated, and an immediate repeat CT thorax revealed PM and bilateral minimal PTX. The possible mechanism may be both the Macklin phenomenon and P-SILI. Cough is known to cause sudden transient alveolar overdistension that occasionally results in alveolar rupture. Thus, it is another risk factor for the development of PM [8]. Two of our patients had complained of cough at presentation. One patient developed PM after 13 days of NIV therapy, signifying increased work of breathing leading to heterogeneous alveolar strain, which may have contributed to alveolar rupture. It is especially relevant in patients in whom lung elastance is unevenly distributed owing to pre-existing emphysematous and/or fibrotic changes. COVID-19 further contributes to the heterogeneity of alveolar stress as it causes a transition from low to high-elastance as a part of the disease process [1]. A recent study by Belletti et al. found that patients with COVID-19 ARDS were at a higher risk for developing PM despite protective mechanical ventilation strategies as compared to patients who had ARDS due to other causes [9]. Moreover, steroids weaken the pulmonary interstitial tissue, which causes alveolar air leak [10]. COVID-19 ARDS increases the risk of pulmonary vascular thrombosis, and subsequent alveolar membrane necrosis and damage [4]. In addition, the liberal promotion of recruitment maneuvers such as proning can potentiate the occurrence of PM and PTX.

In the first wave of the epidemic, early intubation was recommended but with the unprecedented surge and mounting evidence of the association of mortality with invasive ventilation, NIV, HFNC, and other devices were increasingly used for respiratory support. Although NIV may prevent invasive MV, failure of this approach may lead to morbidity and mortality [11]. We hypothesize that the prolonged use of such alternative strategies to delay invasive MV has contributed to PM in our patients.

PM seems to complicate the clinical course and causes a poorer outcome among patients with COVID-19. There are no consensus guidelines in managing COVID-19 patients with pneumomediastinum. Kangas-Dick et al. [12] conducted a retrospective analysis and reported that more than half of the patients with pneumomediastinum were managed conservatively. They also concluded that there was no significant difference in mortality among patients who were managed with tube thoracostomy vis-a-vis those who were managed conservatively. Two of our patients required drainage and two were managed conservatively. A conservative approach is suggested for patients with pneumomediastinum who do not have mediastinal compressive symptoms, although the approach should be individualized and decided on a case-to-case basis.

The physician must be vigilant when choosing to continue oxygen therapy via various oxygen delivery devices in patients with a high respiratory drive as P-SILI can aggravate the disease progression. Also, the tolerance of the patient to NIV and the response to treatment is to be judged. Early use of controlled low tidal volume MV may prevent this progression.

## Conclusions

The occurrence of pneumomediastinum and pneumothorax seems to be multifactorial, the main cause being the structural changes in lungs induced by the virus and inflammation. The second wave of the pandemic has indeed led to an unprecedented surge of patients requiring hospitalization and ICU stay. The shortage of equipment, risk of aerosolization, direct association of mortality with invasive mechanical ventilation resulted in the use of alternative strategies for respiratory support. This potentiated P-SILI inducing altered lung mechanics, progression of the disease, and hence higher incidence of complications like pneumomediastinum and pneumothorax in patients with acute respiratory failure. On account of the ongoing pandemic, the physician must balance the interventions for respiratory support to prevent the occurrence of these events. It is also important to rule out pneumomediastinum and pneumothorax in patients with worsening dyspnea and/or hypoxemia on respiratory support. The outcome of decompression and the conservative approach to pneumomediastinum are similar.
